# Time of day of cardiac surgery and postoperative outcomes in the UK: a secondary analysis of linked national datasets

**DOI:** 10.1111/anae.70125

**Published:** 2026-01-09

**Authors:** Gareth Kitchen, Karen Thomas, Tim Felton, Hannah Durrington, John Blaikley, Stuart W. Grant, David Harrison, Peter McGuigan, Kathryn Rowan, Anthony Wilson, Danny McAuley, Paul Dark

**Affiliations:** ^1^ Division of Immunology, Immunity to Infection and Respiratory Medicine, School of Biological Sciences, Faculty of Biology, Medicine and Health The University of Manchester Oxford Rd Manchester United Kingdom; ^2^ Manchester University NHS Foundation Trust Cobbett House Oxford Rd Manchester United Kingdom; ^3^ The Royal Marsden NHS Foundation Trust London United Kingdom; ^4^ Division of Cardiovascular Sciences, School of Medical Sciences, Faculty of Biology, Medicine and Health The University of Manchester Oxford Rd Manchester United Kingdom; ^5^ Intensive Care National Audit and Research Centre London United Kingdom; ^6^ Regional Intensive Care Unit Royal Victoria Hospital Belfast UK; ^7^ Wellcome‐Wolfson Institute for Experimental Medicine Queen's University Belfast UK; ^8^ Division of Informatics, Imaging and Data Sciences, School of Health Sciences, Faculty of Biology, Medicine and Health University of Manchester Manchester UK; ^9^ Depart of Intensive Care Medicine Northern Care Alliance NHS Foundation Trust Greater Manchester UK

**Keywords:** cardiac surgery, circadian rhythm, peri‐operative outcomes, time of day, time of surgery

## Abstract

**Introduction:**

Uncertainty remains regarding whether the time of day that cardiac surgery is performed affects postoperative outcomes or if the observed variation can be explained by patient or surgical factors.

**Methods:**

A secondary analysis of prospectively collected data was conducted to examine the association between time of cardiac surgery and clinical outcomes. Data were derived from four linked UK datasets: the National Adult Cardiac Surgery Audit; the Case Mix Programme; Hospital Episode Statistics; and Office for National Statistics mortality records. The primary outcomes were hazard of death due to cardiovascular disease and time to hospital readmission for myocardial infarction or acute heart failure. Secondary outcomes included duration of postoperative hospital stay; occurrence of major cardiovascular events; and all‐cause mortality.

**Results:**

Linked data for 24,068 patients were identified. Surgeries performed in late morning (10:00 to 11:59) had the highest mean (SD) predicted risk of death (3.7% (4.6)), compared with 3.2% (3.7) for early morning (07:00 to 09:59), 2.8% (3.4) for early afternoon (12:00 to 13:59) and 3.1% (3.6) for late afternoon (14:00 to 19:59) surgeries, respectively. The primary outcome measures showed an increased hazard of death from cardiovascular disease in the late morning (adjusted hazard ratio 1.18, 95%CI 1.00–1.39), with no difference in hazard of readmission for myocardial infarction or acute heart failure (adjusted hazard ratio 0.97, 95%CI 0.85–1.11). There were no differences in the secondary outcome measures.

**Discussion:**

Time‐of‐day variation in postoperative death due to cardiovascular disease following cardiac surgery was observed, with the highest risk seen in late morning procedures. These findings suggest that intra‐operative or organisational factors specific to this period may influence outcomes. Future research should explore whether individual circadian phenotypes or chronotypes contribute to this variation, supporting a move towards precision and personalised scheduling of cardiac surgery to optimise patient outcomes.

## Plain Language Summary

**What we did:**

We studied health records from over 24,000 people in the UK who had heart surgery. We used information from several large databases to compare what happened to patients depending on what time of day their surgery took place, such as early morning, late morning, or afternoon.

**Why did we do it:**

Some doctors think the time of day of surgery might affect how well patients recover, but it was not clear if this was really true or just linked to other factors like how sick patients were. We wanted to find out if the timing of heart surgery makes a difference to patient outcomes.

**What we found:**

People who had heart surgery in the late morning had a slightly higher risk of dying from heart problems after surgery. The time of surgery did not affect how soon patients were readmitted to hospital for heart attacks or heart failure. There were no differences in how long people stayed in hospital or in other major heart problems. Overall, the study suggests that the time of day—especially late morning—may play a role in recovery after heart surgery. More research is needed to understand why this happens and whether scheduling surgery differently could help improve patient outcomes.

## Introduction

Cardiac surgery and cardiopulmonary bypass create a significant inflammatory stimulus, which is exacerbated further by an ischaemia‐reperfusion insult [1, 2]. This predictable myocardial ischaemia may be linked with less favourable postoperative outcomes [3]. More than 25,000 cardiac surgeries are performed in 37 English, Welsh and Northern Irish hospitals each year, with an overall in‐hospital patient mortality of 2.7% [4]. Inflammatory responses in humans are controlled partially by the circadian clock [5, 6, 7] which plays an important role in outcomes from sepsis [8, 9] and wound healing [10]. The time of day at which inflammatory insults occur can influence patient outcome.

Montaigne et al. studied outcomes following aortic valve replacement surgery and found the incidence of major adverse cardiac events was lower following afternoon surgery compared with the morning [11]. A mechanism mediated through the clock protein REV‐ERBα was suggested. However, multiple subsequent retrospective studies and a meta‐analysis have not replicated this time‐of‐day effect in the outcome of time of cardiac surgery [12, 13, 14, 15, 16]. More recent registry analyses and systematic reviews [12, 15, 17] have reported largely neutral findings, with no consistent morning‐afternoon effect on outcomes. In contrast, a 2024 meta‐analysis across daytime surgeries showed that excess mortality risk was confined to evening and night procedures, rather than within daytime hours [18]. Nevertheless, reviews from the American Heart Association continue to highlight the potential importance of circadian biology in modulating peri‐operative outcomes [19].

Clinical uncertainty remains regarding whether the time of day of cardiac surgery affects postoperative patient outcomes. The aim of this study, therefore, was to investigate the effect of cardiac surgical time of day on postoperative patient outcomes in the UK by performing, for the first time, a secondary analysis of linked national datasets.

## Methods

Data for NHS patients in England, Wales and Northern Ireland were obtained from four national healthcare datasets: the National Adult Cardiac Surgery Audit (NACSA), which collects data on all major cardiac surgery in adults; the Case Mix Programme (CMP), run by the Intensive Care National Audit and Research Centre (ICNARC), which contains data on admissions to adult critical care units; Hospital Episode Statistics (HES), covering all admission episodes in NHS hospitals; and the Office for National Statistics mortality data. The primary data set was the NACSA and records were linked to these securely by researchers at ICNARC using NHS number and date of birth. The NACSA dataset contains data on date, time, type and duration of surgery, pre‐surgery risk factors and immediate complications. The HES dataset contains outcome data (hospital readmissions). The Office for National Statistics dataset provided the date and cause of death and CMP was the central database used for linkage (as part of a wider project researching longer‐term outcomes in critical illness). Data collected for CMP and NACSA undergo extensive validation to assess completeness and consistency before use in audit and research, and there was minimal overlap of data between these databases.

This was a secondary analysis of a linked pseudonymised database from a study of risk modelling for quality improvement in patients who are critically ill. The study received approvals from the Health Research Authority, on the advice of the Confidentiality Advisory Group, for use of patient identifiable data without consent under Section 251 of the NHS Act 2006. National data opt‐outs were applied. Separate research ethics approval for this secondary analysis was not required.

All patients with a record of planned cardiac surgery (elective or urgent) between 1 April 2009 and 31 March 2016 in the NACSA dataset, which could be linked to a related critical care admission in the CMP dataset, and to at least one related hospital admission in the HES dataset, were included. Only the first record of major cardiac surgery for each patient was considered. This analysis occurred before the national data opt‐out on 25 May 2018.

The primary exposure variable of interest was time of knife‐to‐skin during planned cardiac surgery, which was grouped prospectively as: early morning (07:00 to 09:59); late morning (10:00 to 11:59); early afternoon (12:00 to 13:59); and late afternoon (14:00 to 19:59). Patients with surgery starting at any time before 07:00 or after 19:59 were not studied, as they were not considered to be representative of most planned cardiac procedures in UK hospitals. These pragmatic epochs were defined before analysis, with the average cardiac surgery procedure lasting 3–5 h. It is usual in the UK to perform one case in the morning and one in the afternoon [20, 21, 22]. These time periods were divided into a standard and a late start time for morning and afternoon procedures. These pragmatic time periods were chosen so that any difference shown would be translatable to the clinical day.

The primary outcome measures were death due to cardiovascular disease, readmission with myocardial infarction or acute heart failure, and death occurring ≤ 180 days following surgery [11]. Secondary outcome measures were duration of postoperative hospital stay; occurrence of any major cardiovascular event (excluding hospital readmission for myocardial infarction); and all‐cause mortality.

Cox proportional hazards regression was used to examine the association between time of surgery and the hazard of cardiovascular death and hospital readmission for myocardial infarction or acute heart failure. Models were adjusted for pre‐operative risk of death, estimated using the European System for Cardiac Operative Risk Evaluation 2 (EuroSCORE 2), and for duration of surgery (total cross‐clamp time). Adjusted hazard ratios (HRs) with 95%CIs were calculated for the overall cohort and for pre‐specified subgroups defined by procedure type. The same approach was applied to other time‐to‐event secondary outcomes. For the duration of hospital stay, a right‐skewed distribution was corrected using natural logarithmic transformation and linear regression was used to compare adjusted mean differences across time‐of‐day groups, controlling for EuroSCORE 2 and duration of surgery.

Missing data for either EuroSCORE 2 or duration of surgery were addressed using multiple imputation, with estimates from five imputed datasets combined using Rubin's rules. EuroSCORE 2 was chosen a priori as the adjustment measure, as it encompasses major predictors of peri‐operative mortality (age, sex, diabetes, urgency and comorbidity), thereby avoiding collinearity and model overfitting. For all time‐to‐event analyses, patients were censored at the last recorded hospital admission within the HES dataset or at the time of non‐cardiovascular death (where relevant).

## Results

**Figure 1:**
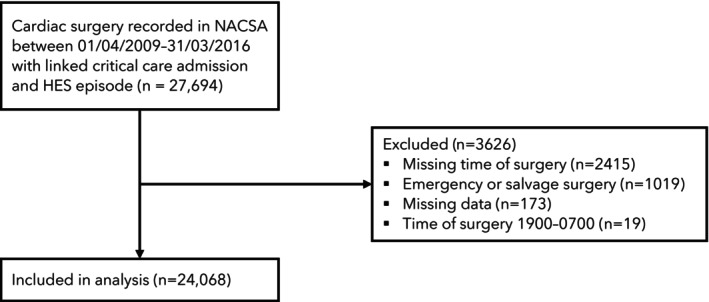
Study flow diagram.

A total of 24,068 patients (Table 1) were included in the analysis (Fig. 1). The most common surgical start time was early morning (07:00–09:59), accounting for 47% of all surgeries. Complete data for EuroSCORE 2 mortality risk were available for 84% of patients, and duration of surgery was recorded for 93%, with comparable levels of missingness across the four time‐of‐day groups. The predicted risk of death was highest among patients undergoing surgery in the late morning (mean (SD) 3.7% (4.6%) compared with 3.2% (3.7%) for early morning, 2.8% (3.4%) early afternoon and 3.1% (3.6%) late afternoon, surgeries, respectively) (Table 2).

**Table 1:** Baseline and pre‐operative characteristics of patients undergoing major elective cardiac surgery between 2009 and 2016, stratified by knife‐to‐skin start time. Values are mean (SD) or number (proportion).

	Early morning	Late morning	Early afternoon	Late afternoon	All patients
	07:00–09:59	10:00–11:59	12:00–13:59	14:00–19:59	
	n = 11,369	n = 1966	n = 6168	n = 4565	n = 24,068
Age; y	68 (11.0)	67 (11.8)	68 (11.0)	68 (10.9)	68 (11.0)
Creatinine; μmol.l^‐1^	95 (50.0)	98 (53.7)	95 (52.4)	95 (50.7)	95 (51.0)
EuroSCORE 2 predicted mortality; %	3.2 (3.7)	3.7 (4.6)	2.8 (3.2)	2.8 (3.4)	3.1 (3.6)
Female sex	3187 (28%)	603 (31%)	1659 (27%)	1259 (28%)	6708 (28%)
Previous cardiac surgery	529 (5%)	139 (7%)	190 (3%)	95 (2%)	953 (4%)
Diabetes mellitus	2563 (23%)	452 (23%)	1428 (23%)	1061 (23%)	5504 (23%)
Hypertension	7736 (68%)	1319 (67%)	4386 (71%)	3170 (70%)	16,611 (69%)
Chronic obstructive pulmonary disease or asthma	1762 (16%)	318 (16%)	922 (15%)	706 (16%)	3708 (15%)
Peripheral vascular disease	1296 (11%)	240 (12%)	634 (10%)	466 (10%)	2636 (11%)
Atrial fibrillation/flutter	1429 (13%)	246 (13%)	791 (13%)	524 (12%)	2990 (12%)
Left ventricular ejection fraction <30%	684 (6%)	119 (6%)	295 (5%)	283 (6%)	1381 (6%)

**Table 2:** Surgical characteristics of the study cohort, grouped by knife‐to‐skin start time. Cohorts were determined prospectively to reflect pragmatic morning and afternoon operating lists. Surgeries commencing before 07:00 or after 19:59 were not included. Values are mean (SD) or number (proportion).

	Early morning	Late morning	Early afternoon	Late afternoon	All patients
	07:00–09:59	10:00–11:59	12:00–13:59	14:00–19:59	n = 24,068
Cross‐clamp time; min	68 (40.8)	75 (52.9)	65 (38.0)	60 (33.0)	67 (40.1)
Urgent cases	3547 (31%)	561 (29%)	1809 (29%)	1428 (31%)	7345 (31%)
Isolated coronary artery bypass graft(s)	5326 (47%)	790 (40%)	3116 (51%)	2411 (53%)	11,643 (48%)
Isolated aortic valve surgery	1933 (17%)	323 (16%)	979 (16%)	962 (21%)	4197 (17%)
Isolated mitral valve surgery	1089 (10%)	211 (11%)	548 (9%)	326 (7%)	2174 (9%)
Non‐isolated procedure	2718 (24%)	509 (26%)	1372 (22%)	750 (16%)	5349 (22%)

After adjustment for patient and surgical factors, the risk of death due to cardiovascular disease was higher in the late morning surgery group compared with early morning surgery (adjusted HR 1.18, 95%CI 1.00–1.39). There was no difference in the risk of hospital readmission for myocardial infarction or acute heart failure (adjusted HR 0.97, 95%CI 0.85–1.11).

Adjusted analyses showed no variation by time of surgery for any of the secondary outcomes, including duration of postoperative hospital stay; occurrence of major cardiovascular events (excluding readmission for myocardial infarction); or all‐cause mortality (Table 3). Duration of hospital stay was consistent across groups, and adjusted mean differences ranged from ‐0.84 to 0.81 days (Table 3). Similarly, adjusted HRs for all‐cause mortality (1.08, 95%CI 0.96–1.22) and major adverse cardiovascular events (1.03, 95%CI 0.93–1.14) indicated no significant association with the timing of surgery.

**Table 3:** Unadjusted and adjusted outcomes following cardiac surgery according to knife‐to‐skin start time. Adjusted hazard ratios (HR) are derived from Cox regression models correcting for Euroscore 2 predicted mortality risk and cumulative cross‐clamp time, with missing baseline data imputed according to the pre‐specified statistical analysis plan. Values are number (proportion) or mean (SD).

	Early morning	Late morning	Early afternoon	Late afternoon	All patients
	07:00–09:59	10:00–11:59	12:00–13:59	14:00–19:59	n = 24,068
Death due to cardiovascular disease					
Events	1098 (10%)	248 (13%)	596 (10%)	403 (9%)	2345 (10%)
Unadjusted HR (95%CI)	Reference	1.39 (1.21–1.59)	0.97 (0.88–1.07)	0.93 (0.83–1.04)	
Adjusted HR (95%CI)	Reference	1.18 (1.00–1.39)	1.09 (0.96–1.23)	1.12 (0.98–1.28)	
Hospital readmission for myocardial infarction or acute heart failure					
Events	1878 (17%)	336 (17%)	1070 (17%)	697 (15%)	3981 (17%)
Unadjusted HR (95%CI)	Reference	1.07 (0.95–1.20)	1.05 (0.97–1.13)	0.91 (0.84–0.99)	
Adjusted HR (95%CI)	Reference	0.97 (0.85–1.11)	1.08 (0.99–1.18)	1.01 (0.91–1.12)	
Length of stay in an acute hospital					
Duration of hospital stay; days	14.7 (15.6)	15.8 (17.1)	14.0 (15.5)	13.9 (14.1)	14.4 (15.4)
Mean difference	Reference	1.2 (0.4–2.0)	‐0.7 (‐1.2 to ‐0.2)	‐0.8 (‐1.3 to ‐0.3)	
Adjusted mean difference	Reference	0.02 (‐0.78–0.81)	‐0.31 (‐0.84–0.21)	0.25 (‐0.28–0.78)	
Cardiovascular death or hospital readmission for acute heart failure					
Events	2370 (21%)	478 (24%)	1326 (22%)	880 (19%)	5054 (21%)
Unadjusted HR (95%CI)	Reference	1.22 (1.11–1.35)	1.02 (0.95–1.09)	0.92 (0.85–0.99)	
Adjusted HR (95%CI)	Reference	1.07 (0.95–1.20)	1.08 (1.00–1.18)	1.07 (0.98–1.17)	
All‐cause mortality					
Events	2313 (20%)	458 (23%)	1224 (20%)	875 (19%)	4870 (20%)
Unadjusted HR (95%CI)	Reference	1.23 (1.12–1.36)	0.93 (0.87–1.00)	0.96 (0.89–1.04)	
Adjusted HR (95%CI)	Reference	1.08 (0.96–1.22)	1.01 (0.93–1.11)	1.08 (0.98–1.18)	
Any major adverse cardiovascular event					
Events	3364 (30%)	633 (32%)	1844 (30%)	1273 (28%)	7114 (30%)
Unadjusted HR (95%CI)	Reference	1.15 (1.05–1.25)	0.99 (0.94–1.05)	0.94 (0.88–1.00)	
Adjusted HR (95%CI)	Reference	1.03 (0.93–1.14)	1.05 (0.98–1.13)	1.06 (0.98–1.14)	

## Discussion

In this large UK‐wide cohort of 24,068 cardiac surgeries, we observed a modest increase in cardiovascular mortality among patients whose surgery commenced in the late morning (10:00–11:59). This persisted after adjustment for pre‐operative risk and duration of surgery, without corresponding increases in myocardial infarction, readmission for heart failure or all‐cause mortality. These findings suggest that even within routine daytime hours, surgical timing may influence cardiovascular outcomes, meriting further investigation of biological and system‐level mechanisms.

Circadian rhythm, a fundamental biological process, exhibits remarkable evolutionary conservation, spanning the ancient cyanobacteria that oxygenated the atmosphere of Earth to complex mammalian systems, including humans [7, 11, 23]. This daily cyclical variation, generated by the 24‐h rotation of the Earth, is controlled mechanistically by the circadian clock, a core set of genes present in almost every cell in the human body, including cardiac myocytes. One proposed mechanism is diurnal variation in myocardial tolerance to ischaemia–reperfusion injury, modulated by the intrinsic cardiac clock [24].

Our findings contribute to a mixed body of evidence regarding time‐of‐day effects in cardiac surgery. Montaigne et al. reported lower peri‐operative myocardial injury in patients undergoing afternoon aortic valve surgery, mediated by transcriptional activation of circadian clock genes [11]. However, their single‐centre randomised trial was small and may lack generalisability. In contrast, Fudulu et al. found no significant morning–afternoon differences in outcomes or myocardial injury using both systematic review and UK NACSA data [12]. Interpretation is, however, constrained by study heterogeneity and variable endpoint definitions.

The effect of surgical timing on daytime surgery outcomes is a topic of significant interest within the healthcare provider community, largely because adjusting the time of incision represents a straightforward and low‐cost intervention. The systematic review by Meewisse et al. showed an elevated mortality risk for elective surgeries performed during evening and night hours compared with daytime [18]. In the UK, due to the large datasets available and the ability to link these using the NHS patient number, we are in a unique position to address the question of the impact of time of cardiac surgery on outcome.

**Figure 2:**
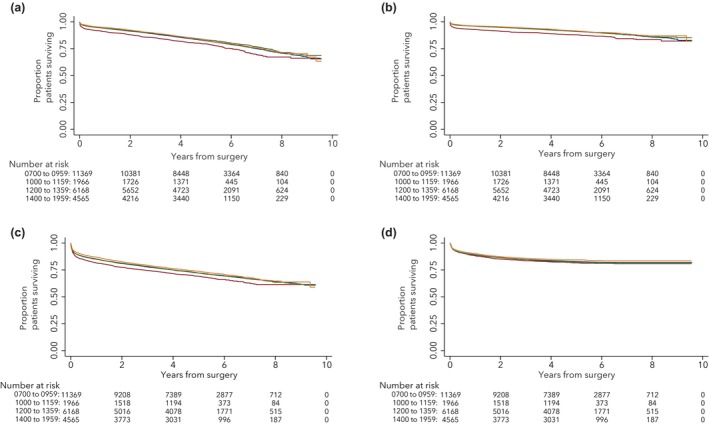
Time‐to‐event curves for outcomes following cardiac surgery by time of day. Kaplan–Meier curves showing time from surgery to: (a) death due to cardiovascular disease; (b) any major adverse cardiovascular event; (c) hospital readmission for myocardial infarction or acute heart failure; and (d) all‐cause mortality. Patients were grouped by knife‐to‐skin start time: blue, early morning; red, late morning; green, early afternoon; and orange, late afternoon.

Our results reveal a modest signal of increased cardiovascular mortality associated with cardiac surgery performed between 10:00 and 11:59, when compared with surgery performed between 07:00 and 09:59. Inspection of the Kaplan–Meier curves (Fig. 2) shows that divergence between the late and early morning groups emerges within the first postoperative months. This early separation suggests that any excess cardiovascular mortality is likely driven by peri‐operative factors rather than long‐term disease progression. No increase was observed in the early or late afternoon groups, a pattern partially consistent with Montaigne et al. [11]. Although absolute risk differences are small, given the > 25,000 annual UK procedures, these findings could translate into meaningful population‐level effects.

The research benefits from several strengths. First, it offers a UK‐wide perspective, leveraging comprehensive data linkage across a large patient population. Second, it utilised high‐quality, nationally audited datasets, ensuring data accuracy through meticulous cleaning and preparation by trained data clerks. Finally, the study attempted to account for potential confounders by adjusting outcomes for peri‐operative risk of death using EuroSCORE 2, which incorporates key mortality predictors such as age, sex, diabetes and urgency of surgery, enhancing the reliability of these findings.

We are cognisant of the limitations inherent in our retrospective study design and recognise that causal inference cannot be made. The need for data imputation, potential bias from patient loss during the linkage process and the absence of adjustments for seasonality and inter‐centre variability in surgical practice are also limitations. While the UK‐wide data provides a valuable national perspective, its generalisability to other healthcare systems is limited by potential differences in surgical protocols and patient populations. Residual confounding remains possible, although EuroSCORE 2 incorporates age, sex, diabetes, and surgical urgency, and baseline comparisons revealed no major group differences. We selected EuroSCORE 2 as the principal adjustment variable a priori because it integrates key predictors of peri‐operative mortality within a single validated metric. Including EuroSCORE 2 and any of its constituent variables in a multivariable model would introduce collinearity and risk statistical overfitting without changing the clinical interpretation materially. Furthermore, the dataset, derived from 2016, raises concerns about temporal validity. However, we contend that the data remains relevant. The COVID‐19 pandemic disrupted cardiac surgery significantly, resulting in a reduction of cases and an increase in patient comorbidity and complexity [25]. Given these factors, we assert that our dataset provides the most recent population‐based snapshot of patient cohorts relevant to current clinical practice.

Looking forward, integrating circadian biology into peri‐operative planning could support a more personalised, precision medicine approach, tailoring surgical times to individual chronotypes (‘day birds’ vs. ‘night owls’) [26]. This could be explored through large, prospective trials or population‐scale analyses linking electronic health records with wearable circadian data.

This UK‐wide analysis suggests a modest increase in cardiovascular mortality for late morning cardiac surgery, independent of surgical risk. While exploratory and hypothesis‐generating, these findings highlight the need to examine both surgical and circadian factors influencing surgical outcomes. Future studies integrating circadian biology and personalised scheduling may inform precision approaches to optimise peri‐operative care.
